# Micro-hub location selection for sustainable last-mile delivery

**DOI:** 10.1371/journal.pone.0270926

**Published:** 2022-07-05

**Authors:** Michaela Novotná, Libor Švadlenka, Stefan Jovčić, Vladimir Simić

**Affiliations:** 1 Department of Transport Management, Marketing and Logistics, University of Pardubice-Faculty of Transport Engineering, Pardubice, Czech Republic; 2 Department of Transport and Traffic Engineering, University of Belgrade-Faculty of Transport and Traffic Engineering, Belgrade, Republic of Serbia; Universita degli Studi del Molise, ITALY

## Abstract

Sustainable Last-Mile Delivery (LMD) is one of the key phases in city logistics. Micro-hubs in cities are new emerging solutions for an easier and viable last-mile delivery process. The important question in smart and modern cities is the determination of the best micro-hub location for the LMD. This paper solves the micro-hub location selection for sustainable LMD using the multi-criteria decision-making (MCDM) techniques. The main reason for solving the micro-hub location selection is to make the last-mile delivery process in Pardubice as easier and effortless as possible. The Best-Worst Method (BWM), Criteria Importance Through Intercriteria Correlation (CRITIC) method, and Weighted Aggregated Sum Product Assessment (WASPAS) method are coupled to solve the micro-hub location selection for sustainable LMD. First, five criteria and alternatives are identified and discussed with the experts. Second, the hybrid criteria importance is determined by combining the BWM and CRITIC methods. Third, the obtained hybrid weights are integrated within the WASPAS method to rank the micro-hub locations. The findings of the Hybrid BWM-CRITIC-WASPAS model show the Alternative 2 („Hůrka“) as the best possible location for Pardubice in the context of the LMD. In addition, a comparative analysis with some of the existing MCDM approaches is conducted for the same problem and its results show a high level of matching with the applied hybrid BWM-CRITIC-WASPAS method, which means that Alternative 2 („Hůrka“) is strongly recommended as a micro-hub location for sustainable LMD in Pardubice.

## 1. Introduction

Cargo transportation plays an important role in the national and international trade [1]. For improved functioning of a city, efficient and well-planned transport is one crucial fact [2]. According to Muñoz-Villamizar et al. [3], the last-Mile Deliveries (LMDs) had become essential for customers in cities, making city centers one of the most complex areas. Every postal company, whether it is a private or public postal operator, is based on a branched network throughout the territory where it provides services [4]. In recent years, the issue of last-mile delivery (LMD) has been gaining particular attention by both Scientists and National Postal Operators (NPOs). Due to the rapid development of E-commerce as well as increased customers’ demands, National Postal Operators are under enormous pressure in terms of last-mile delivery (LMD). Along with it, the COVID-19 crisis has further hampered the business of NPSs. Simić et al. [5] emphasized that the delivery system had been particularly affected during the COVID-19 (coronavirus) outbreak when a large part of the world population encountered some level of restricted movement and social interactions. In response to the COVID-19 crisis, various LMD modes and solutions have emerged. To have a sustainable delivery process, various possible solutions such as Cargo Bikes, Parcel lockers, micro-hubs, etc., are appearing. The European Commission’s document [6] emphasized that at least 30 million zero-emmision vehicles would be in operation on European roads by 2030. In addition, automated mobility would be deployed at large scale. When it comes to the last-mile delivery process in urban areas, this is a really important question for the National Postal Operators’ management and other private postal operators. A special attention should be placed to all those future transportation issues. In addition, Hruška et al. [7] and Hruška et al. [8] emphasized that it was necessary to increase flexibility to respond to rapidly changing markets. Deveci et al. [9] emphasized that climate change affected and reshaped everything, from economics to politics, and to achieve successful results, especially in the transportation sector, cooperation was the key factor. In the same year, Deveci et al. [10] stated that the facility location selection problem had attracted a large interest of researchers in recent years. Akyrt et al. [11] proposed the methodology to solve the location selection problem for a flight base. Deveci et al. [12] decided on the floating offshore wind farm site selection in Norway.

This paper aims at solving the micro-hub location selection problem for the last-mile delivery process in the context of Pardubice. Micro-hub should be defined as a location in the territory of the city where the postal network operators should consolidate the shipments and from that location to deliver them to the customers’ addresses by cargo bikes. There are two stages of the delivery process. The first one is the shipment consolidation process of all postal operators, the national and the private ones, at the established micro-hub location. The second one is the last-mile delivery process to the final customers by Cargo Bikes. According to the authors’ knowledge and reviewed literature, there is no paper dealing with the micro-hub location selection in the context of Pardubice city, which should be emphasized as one of the research gaps. The micro-hub location selection problem is not so easy task for the postal operators since multiple interrelated criteria affect the decision-making process. This is a typical kind of a multi-criteria decision-making problem since multiple conflicting criteria affect the micro-hub location selection such as area, cargo bike availability, costs, etc. It is important to emphasize that those multiple criteria do not have the same importance in a decision-making process.

There are two of the research questions emerging in the context of micro-hub location selection considered in this paper.

The first one is the determination of the criteria importance for the micro-hub location selection for the sustainable last-mile delivery process. There are several articles found in the literature dealing with the facility location problem. For example, Rosenberg et al. [13] investigated the success of the micro-depot network for LMD considering the economic, social, and environmental criteria. Awasthi et al. [14] applied the fuzzy TOPSIS method to locate the urban distribution centers. They considered criteria such as accessibility, security, costs, environmental impact, proximity to customers and suppliers, resource availability, quality of service, the possibility of expansion, and conformance to sustainable freight regulations. Janjević et al. [15] developed a model for strategic scenarios evaluation for sustainable urban distribution using Urban Consolidation Centers (UCCs) in Brussels. They considered several scenarios and included geographical aspects, a fleet of UCCs vehicles, operation hours of the UCC, pricing of the UCC services, etc. Arrieta-Prieto et al. [16] used the criteria such as layout dimensions, number of carriers, area, demand, and payload per truck to locate an urban micro-consolidation center. In this paper, to find the best micro-hub location, we considered the criteria such as sum of distances from sorting center, area, cargo bike availability, cycle distance and costs.

The second research question is the determination of the best possible micro-hub location in the Pardubice city according to the established criteria. To fill the research gap and answer the research questions mentioned above, the authors of this article propose the hybrid BWM-CRITIC-WASPAS approach. The hybrid method is the combination of two methods used to obtain the criteria weights that are necessary for the micro-hub location selection. Namely, the Best-Worst method (BWM) and the CRiteria Importance through Inter-criteria Correlation (CRITIC) method were coupled and the hybrid criteria weights were identified. The main reason for coupling those two methods lies in the fact that the combination of the subjective (Best-Worst Method) and objective (CRITIC method) methods would obtain stronger criteria weights and give more reliable results since the CRITIC helps to eliminate subjectivity in a decision-making process, while the best-worst method helps to prioritize the best and the worst criterion. The WASPAS method is, on the other side, a combination of the two verified methods, such as the Weighted Sum Method (WSM) and the Weighted Product Method (WPM). This method is relatively new in the scientific literature and has been proven as a very effective one in solving the multi-criteria decision-making problems in various fields such as Manufacturing Industry [17], Logistics Industry [18], Digital Library [19], Postal Industry [5], etc.

However, the main limitations of the BWM-CRITIC-WASPAS methods are that they mostly deal with the crisp values and don’t deal with uncertainty.

The main contribution of this article is twofold: methodological and practical. When it comes to methodological contributions, it should be stated that for the first time the hybrid BWM-CRITIC-WASPAS approach was utilized to solve the micro-hub location selection. The proposed methodology has its practical use since it was applied to a real-life case study to solve the micro-hub location selection in the context of Pardubice city. The hybrid BWM-CRITIC-WASPAS method was compared to the other MCDM approaches to show its stability. In addition, the hybrid BWM-CRITIC-WASPAS method can be applied to any other MCDM city logistics problem considering many alternatives and including multiple conflicting criteria.

The rest of the paper is organized in the following manner: Section 2 presents the literature review of the existing state-of-the-art. Section 3 describes the methods used to solve the micro-hub location selection problem. The application of the methodology in the context of Pardubice City is presented in Section 4. Section 5 presents the results obtained, while Section 6 gives the concluding remarks and future directions and recommendations.

## 2. Literature review

The literature review is organized into three sub-sections. The first sub-section overviews the current state-of-the-art in City logistics, especially in the last-mile delivery. The second sub-section reviews the applications of the Best-Worst Method (BWM), CRITIC, as well as the WASPAS method. The last sub-section points out the research gaps of those methods.

### 2.1 Overview of current state-of-the-art in city logistics

In recent years, the field of City Logistics has been becoming more and more popular and gains special attention from both practitioners and scientists. Three electronic databases such as Google Scholar, Scopus and Web of Science were the main sources for the exploration of the current state-of-the-art City Logistics field. Various City logistics problems, especially the ones that consider the last-mile delivery process, have been found and briefly described in Table 1.

**Table 1:** Review on the last-mile delivery in the last decade.

Author (Publication Year)	Problem considered
Cetin and Gencer [20]	VRP with hard-time windows and simultaneous pickup and delivery
Çatay [21]	A new saving-based ant algorithm for the Vehicle Routing Problem with Simultaneous Pickup and Delivery
Petersen and Ropke [22]	Pickup and Delivery Problem with Cross-Docking Opportunity
Grzybowska and Barceló [23]	Decision Support System (DSS) for real time freight management
Klumpp et al. [24]	Critical information and process requirements that retailers and logistics service providers face in daily operations by using electric vehicles for last-mile distribution.
Cleophas and Ehmke [25]	Profitability of the delivery
Muñoz-Villamizar et al. [26]	Collaborative to non-collaborative last-mile delivery in urban systems with stochastic demands in Colombia
Faccio and Gamberi [27]	Distribution problem from a sustainable perspective in Italy
Wu et al. [28]	Locating self-collection points for last-mile logistics
Nguyen et al. [29]	Multi-trip pickup and delivery problem with time windows and synchronization
Manier et al. [30]	Pickup and delivery problems with multiple time windows (PDPMTW) with paired demands
Chami et al. [31]	Bi-objective selective pickup and delivery problem with time windows and paired demands
Butrina et al. [32]	Key Factors in Urban Pickup and Delivery of Goods
Lu and Yang [33]	Hybrid route planning approach for logistics with pickup and delivery
Bettinelli et al. [34]	Multi-Trip Separate Pickup and Delivery Problem with Time Windows at Customers and Facilities
Wang et al. [35]	Collaborative Mechanism for Pickup and Delivery Problems with Heterogeneous Vehicles
Guo et al. [36]	Simulation study about crowdsourced delivery in last-mile logistics
Leyerer et al. [37]	Innovative parcel delivery concept for last-mile delivery operations to support tactical planning decisions
Wang et al. [38]	Advances of the ride sharing in the last-mile parcel delivery mechanism
De Mello Bandeira [39]	Simulation study about crowdsourced delivery in last-mile logistics.
Perboli and Rosano [40]	Parcel delivery in urban areas
Oliveira et al. [41]	Accessibility from collection and delivery points towards the sustainability of the e-commerce delivery
Bergman et al. [42]	City logistics distribution problem by integrating first-mile pickup and last-mile delivery
Yu et al. [43]	Online pickup and delivery problem with constrained capacity to minimize latency
McLeod et al. [44]	Potential environmental and financial benefits of switching from traditional van-based deliveries to an alternative operating model
De Kervenoael et al. [45]	Engagement of delivery workers in urban last mile delivery for sustainable logistics
Souza et al. [46]	How the new forms of operation could be implemented in developing countries for the cities
Kirschstein [47]	Energy Consumption model for drones
*Our study*	*Micro-hub Selection for Sustainable Last-Mile Delivery*: *A case study of Pardubice*

As can be noticed from Table 1, the micro-hub location selection problem for last-mile delivery has not been previously solved, especially not for the Pardubice city. To fill this gap, the authors of this paper coupled the Best-Worst and CRITIC methods with the WASPAS one. The result is the optimal micro-hub location for the last-mile delivery in Pardubice city. The main reason for using the best-worst method is because this method has been proven as a very effective one in many MCDM research studies [48–51]. The CRITIC method is applied in this paper because of its usefulness in obtaining the objective criteria weights, while the subjectivity is eliminated.

### 2.2 BWM, CRITIC and WASPAS methods–Overview of applications

This sub-section reveals an extensive review of the existing state-of-the-art applications of the Best-Worst, CRITIC, and WASPAS methods.

Best-Worst Method (BWM) is a multi-criteria decision-making (MCDM) method developed by Rezaei [52]. This method is mainly used to obtain the criteria’ importance in the MCDM problems. Various applications of the Best-Worst method are summarized in Table 2.

**Table 2:** Applications of the Best-Worst Method (BWM).

*Authors (Publication year)*	*Problem considered*
Ortega et al. [53]	Sustainable Park and Ride facility location
Pamučar et al. [50]	Renewable Energy
Zhou et al. [54]	Evaluation of urban photovoltaic charging station in Beijing
Moslem et al. [51]	Mobility choice after COVID-19 in Italy
Kant and Gupta [55]	Sustainable Urban Freight Strategies in Jaipur city in India
Ozmen and Aydogan [49]	Logistics Center Location
Ali and Rashid [56]	Robot Selection Process
Sarubi [57]	Mining Equipment Manufacturing
Mahmoudi [58]	Multiple Experts MCDM problem under uncertainty
Duleba et al. [59]	Commuting Modal Spit
Rodríguez-Gutiérez et al. [48]	SMEs Under Sustainability Perspective
Guler and Yomralioglu [60]	Bicycle Station and Lane Location Selection
Gholamreza et al. [61]	Green Supplier Selection and Supplier Development segmentation
Ma et al. [62]	Evaluation of the Locations for Smart Waste Bins

Criteria Importance through Inter-criteria Correlation (CRITIC) method is another method for obtaining the objective criteria weights. This method is proposed by Diakoulaki et al. [63]. The obtained criteria weights by this method include both contrast intensity of each criterion and conflict between criteria. Various applications of the CRITIC method should be found in the scientific literature. The applications of the CRITIC method are summarized in Table 3.

**Table 3:** Applications of the CRITIC method.

Authors (Publication year)	Problem considered
Ghorabaee et al. [64]	Third-Party Logistics (3PL) Provider Assessment
Adali and Işık [65]	Contract Manufacturers Selection Problem
Xu and Chen [66]	Evaluation on Financial Performance of Real Estate Companies
Ghorabaee et al. [67]	Evaluation of construction equipment
Tuş and Adalı [68]	Software selection problem
Adalı et al. [69]	Hospital Site Selection
Liaw et al. [70]	Evaluation of outsourcing providers in Manufacturing
Shi et al. [71]	Comprehensive power quality evaluation of microgrid
Zafar et al. [72]	Block-chain evaluation system
Krishman et al. [73]	Smart Phone Selection
Gupta et al. [74]	Assessment of stress level in urban area’s during COVID-19 outbreak

The Weighted Aggregated Sum Product Assessment (WASPAS) method is a simple coupling of both the Weighted Sum Method (WSM) and the Weighted Product Method (WPM). The WASPAS method is originally developed by Zavadskas et al. [75], and since that time, there are many applications and extensions of this method that should be noticed in the scientific literature. An extensive overview of the applications of the WASPAS method either separately or coupled with other ones is summarized in Table 4.

**Table 4:** Applications of the WASPAS method.

Authors (Publication year)	Problem considered
Shankar and Zavadskas [17]	Manufacturing Industry
Karabašević et al. [76]	Personnel selection
Ghorabaee et al. [77]	Evaluation of green suppliers
Jayant and Singh [18]	3PL provider selection
Ilbahar and Kahraman [78]	Retail store performance measurement
Mathew and Sahu [79]	Conveyor and automated guided vehicle selection
Stojić et al. [80]; Petrović et al. [81]	Supplier selection
Tuş and Adalı [68]	Software selection
Badalpur and Nurbakhsh [82]	Risk analysis of a road construction project
Mesran et al. [83]	Ranking teacher performance
Yörükoğlu and Aydın [19]	Digital Library Evaluation
Miҫ and Antmen [84]	University Location Selection
Simić et al. [5]	Last-mile delivery selection mode
Yuan and Wu [85]	Vibro-diagnostic models for rotational machines
Yücenur and Ipekçi [86]	Marine current energy plant location selection
Ma et al. [62]	Evaluation of the Locations for Smart Waste Bins

When it comes to the location selection problem in various fields, except the BWM, CRITIC, and WASPAS methods, there are many papers in the scientific literature using the MCDM methods. For example, Chou [87] applied the fuzzy MCDM methodology to find the best hub location in the marine transportation in southeastern Asia. Ding [88] conducted the fuzzy MCDM approach to select a Hub Location for Global Shipping Carrier. Brito and Botter [89] analyzed the feasibility of a global logistics hub in Panama by using the AHP method. Amin et al. [90] solved the warehouse selection problem by utilizing the AHP and TOPSIS methods. Özkurt and Figen [91] coupled fuzzy logic with the TOPSIS method to identify the healthcare location for a regional hospital. Karaşan et al. [92] investigated the best location for the electric vehicle charging stations in Turkey, by utilizing the fuzzy logic, DEMATEL, and AHP methods. Guler and Yomralioglu [60] solved the problem of location selection for the electric vehicle charging stations by coupling the GIS tools, fuzzy-AHP, and TOPSIS methods. The fuzzy-AHP and TOPSIS are also utilized by Cinar [93] for bank branch location selection. Ugo [94] applied the fuzzy-TOPSIS approach to finding the best location for the residential base camp. In this paper, we apply the hybrid BWM-CRITIC-WASPAS method to find the best micro-hub location for sustainable LMD in Pardubice.

### 2.3 Research gaps

As can be seen from Tables 2–4, the micro-hub location selection problem has been solved neither by using separate methods (Best-Worst, CRITIC, and WASPAS) nor combined. Therefore, to fill this major gap, with the objective of the combined hybrid BWM-CRITIC-WASPAS, this research paper solves the micro-hub location selection problem in the context of Pardubice.

## 3. Methodology

This section gives some preliminaries and presents the integrated hybrid BWM-CRITIC-WASPAS decision-making model for micro-hub location selection in Pardubice. Three experts from the field of postal traffic and logistics have been involved in the micro-hub location selection in Pardubice. They were interviewed by telephone since it was recommended due to the COVID-19 pandemic. Personal information such as the names and surnames of the experts is not presented in the paper since they keep their privacy. However, a piece of information that the experts revealed is that Expert 1 has a Ph.D. in the postal branch with eight years of experience in distribution, Expert 2 is the associate professor at the Postal Traffic Department with twelve years of experience, while Expert 3 is the associate professor at the Department of Postal Traffic and Logistics with ten years of experience. In this way, the study met relevant personnel data privacy laws.

### 3.1 Best-worst method

The Best-Worst method is one of many MCDM methods originally developed by Rezaei [52]. This method is meant to be used in obtaining the criteria’ importance. The fundamental idea of this method, according to Rezaei [52], is when making a pairwise comparison *a*_*ij*_, both the direction and the strength of the preference *I* over *j* are considered by the decision-maker. Nevertheless, elaborating the strength of the preference is not so easy task for decision-makers since inconsistency is always presented. The same author also emphasized that when executing a pairwise comparison *a*_*ij*_, the decision-maker expressed both the direction and the strength of the preference *i* over *j*. Mostly, the decision-maker has no problem in expressing the direction.

If we had a certain number of *n* criteria and want to make a comparison between them on a scale from 1/9 to 9, we would obtain the resulting matrix:

$$A=[\begin{array}{cccc} a_{11} & a_{12} & \cdots & a_{1n}\\ a_{21} & a_{22} & \cdots & a_{2n}\\ \vdots & \vdots & \vdots & \vdots \\ a_{n}{}_{1} & a_{n2} & \cdots & a_{nn} \end{array}];$$

Where *a*_*ij*_ expresses the relative preference of criterion *i* to criterion *j*. If *a*_*ij*_ is equal to 1, it means that both criteria *i* and *j* have the same importance. If *a*_*ij*_ is higher than 1, it means that criterion *i* is more important than criterion *j*. For instance, let us assume that *a*_*ij*_ = 9. It shows the extreme importance of *i* to *j*. On the contrary, the importance of *j* to *i* is expressed by *a*_*ji*_. If criterion *i* is three times more important than criterion *j*, it means that *a*_*ij*_ = 3 while on the other side *a*_*ji*_ = 1/3.

After a short introduction, Rezaei [52] described the Best-Worst method through the following steps:

**Step 1.** Determine a set of decision criteria.

The criteria {*c*_1_, *c*_2_,…*c*_*n*_} are determined be used to make a desirable decision.

**Step 2.** Determine the best (e.g., most desirable, most important) and the worst (e.g., less desirable, less important) criterion.

In the case that more than one criterion is the best or the worst one, one of them can be chosen arbitrarily. At this stage, the decision-maker identifies the best and the worst criterion in general. At this stage, no comparison is performed.

**Step 3.** Determine the preference of the best criterion to all the other ones using a scale between 1 and 9.

The resulting Best-to-Others vector should be defined:

$$A_{B}=(a_{B1},a_{B2,\ldots }a_{Bn})$$ (1)

Where *a*_*Bj*_ shows the preference of the best criterion *B* over criterion *j*. So, it is clear that *a*_*BB*_ = 1.

**Step 4.** Determine the preference of all the criteria to the worst one using a scale between 1 and 9.

The resulting Others-to-Worst vector should be defined:

$$A_{w}={\left(a_{1w},a_{2w,\ldots }a_{nw}\right)}^{T}$$ (2)

Where *a*_*jw*_ indicates the preference of the criterion *j* to the worst one *W*. It is clear that *a*_*ww*_ = 1.

**Step 5.** Find the optimal weights ($w_{1}^{*},w_{2}^{*},\ldots ,w_{n}^{*}$)

The optimal weight for the criteria is the one where, for each pair of *W*_*B*_/*W*_*j*_ and *W*_*j*_/*W*_*w*_, we have *W*_*B*_/*W*_*j*_ = *a*_*Bj*_ and *W*_*j*_/*W*_*w*_ = *a*_*jw*_. To fulfill these conditions for all *j*, we should find a solution where the maximum absolute differences $\left|\frac{W_{B}}{W_{j}}-a_{Bj}\right|$ and $\left|\frac{W_{j}}{W_{w}}-a_{jw}\right|$ for all *j* is minimized. Considering the non-negativity and sum condition for the weights, that would result in the following problem:

$$min\;\underset{j}{\max}\left\{\left|\frac{W_{B}}{W_{j}}-a_{Bj}|,|\frac{W_{j}}{W_{w}}-a_{jw}\right|\right\}$$ (3)

s.t.


$$\sum_{j}W_{j}=1;\;W_{j}\geq 0\;\mathrm{for}\;\mathrm{all}\;j.$$

After Eq 3, the problem can be reformulated to the following linear programming problem: Min ξ

s.t.


$$\begin{array}{l} |\frac{W_{B}}{W_{j}}-a_{Bj}|\leq \xi ,\;\mathrm{for}\;\mathrm{all}\;j.\\ |\frac{W_{j}}{W_{w}}-a_{jw}|\leq \xi ,\;\mathrm{for}\;\mathrm{all}\;j.\\ \sum_{j}W_{j}=1;\;W_{j}\geq 0\;\mathrm{for}\;\mathrm{all}\;j. \end{array}$$ (4)

By solving Eq 4, the optimal weights ($w_{1}^{*},w_{2}^{*},\ldots ,w_{n}^{*}$) and ξ* are obtained.

The consistency ratio of the model is calculated using the following Equation:

$$Consistency\;Ratio=\frac{\xi }{CI};$$ (5)

where ξ is the optimal objective value of model (5), and CI is the consistency index which can be taken from Table 5.

**Table 5:** Consistency index [52].

*a*_*Bw*_	1	2	3	4	5	6	7	8	9
***Consistency index max* (ξ)**	0.00	0.44	1.00	1.63	2.30	3.00	3.73	4.47	5.23

### 3.2 CRITIC method

Diakoulaki et al. [63] emphasized that in the MCDM problems, the cRiteria Importance Through Inter-criteria Correlation (CRITIC) was an effective method for determining the objective criteria weights. The obtained criteria weights by this method include both contrast intensity of each criterion and conflict between criteria. According to Ghorabaee et al. [64], the contrast intensity of criteria is considered by the standard deviation, and conflict between them is measured by the correlation coefficient. The CRITIC method for obtaining the criteria weights may be presented through the following steps [65]:

**Step 1** is the calculation of the transformations of performance values (**x**_**ij**_) and obtaining criteria vectors. It may be presented through Eq 6:

$$x_{ij}{}^{T}=\{\begin{array}{c} \frac{x_{ij}-x_{j}^{-}}{x_{j}^{*}-x_{j}^{-}}if\;j\;\epsilon \;B;\\ \frac{–x_{ij}}{x_{j}^{-}-–}if\;j\;\epsilon \;N; \end{array}$$ (6)

where: *x*_*ij*_^*T*^ presents the transformed value, x_j_ presents the vector of j-th criterion, $x_{j}^{*}$ and $x_{j}^{-}$ presents the ideal and anti-ideal values with respect to j-th criterion. If j ϵ B then $x_{j}^{*}={max}_{i}x_{ij}$ *and* $x_{j}^{-}={min}_{i}x_{ij}$. If j ϵ N then $x_{j}^{*}={min}_{i}x_{ij}$ *and* $x_{j}^{-}={max}_{i}x_{ij}$.

In **Step 2**, the standard deviation ỽ_j_ of each criterion is calculating using the corresponding vector.

**Step 3** formulates a mxm square matrix R with elements r_jk_, where k = 1,2,…,m.


$$R={\left[r_{jk}\right]}_{mxm};$$ (7)

The elements of this matrix are the linear correlation coefficient between the vectors x_j_ and x_k_.

In **Step 4**, the information measure of each criterion is calculated by applying the Eq (8):

$$H_{j}={ỽ}_{j}\sum_{k=1}^{m}(1-r_{jk});$$ (8)

**Step 5** is the final step, and the criteria weights are calculating here by applying the Equation

(9):

$$W_{j}=\frac{H_{j}}{\sum_{k=1}^{m}H_{k}};$$ (9)

### 3.3 WASPAS method

Weighted Aggregated Sum Product Assessment (WASPAS) method belongs to the multi-criteria decision-making methods (MCDM) and is a relatively new approach introduced by Zavadskas et al. [75]. According to Zavadskas et al. [75], this MCDM method integrates the Weighted Sum Model (WSM) and Weighted Product Model (WPM) for decision-making process. The WASPAS method may be described through four main steps. Let us suppose that *W*_*j*_ denotes the weight of *j*-th criterion and *x*_*ij*_ represents the performance value of *i*-th alternative according to *j*-th criterion (*i* = 1,2,…,*n*; *j* = 1,2…,*m*).

**Step 1.** Obtain linear normalization of performance values.

To obtain linear normalization of performance values, it is necessary to apply the Eq (10):

$${\bar{x}}_{ij}=\{\begin{array}{c} \frac{x_{ij}}{max_{i}x_{ij}}if\;j\;\epsilon \;B;\\ \frac{min_{i}x_{ij}}{x_{ij}}if\;j\;\epsilon \;N; \end{array}$$ (10)

where: *B* and *N* represent the sets of beneficial and non-beneficial criteria, respectively.

**Step 2.** Calculate the measures of WSM $Q_{i}^{\left(1\right)}$ and WPM $Q_{i}^{\left(2\right)}$ for each alternative (Eq (11) and Eq (12)):

$$Q_{i}^{\left(1\right)}=\sum_{j=1}^{m}W_{j}\cdot {\bar{x}}_{ij};$$ (11)

$$Q_{i}^{\left(2\right)}=\prod_{j=1}^{m}{\left({\bar{x}}_{ij}\right|}^{W_{j}};$$ (12)

**Step 3.** Calculate the aggregated measure of the WASPAS method for each alternative as follows (Eq 13).

$$Q_{i}=\lambda Q_{i}^{\left(1\right)}+(1-\lambda )Q_{i}^{\left(2\right)};$$ (13)

where: *λ* is the parameter of the WASPAS method and could be changed in the range of 0 to 1. When *λ* = 1, the WASPAS method is transformed to WSM, and *λ* = 0 leads to WPM model.

## 4. Application of the methodology to the micro-hub location selection in Pardubice city

**Fig 1:**
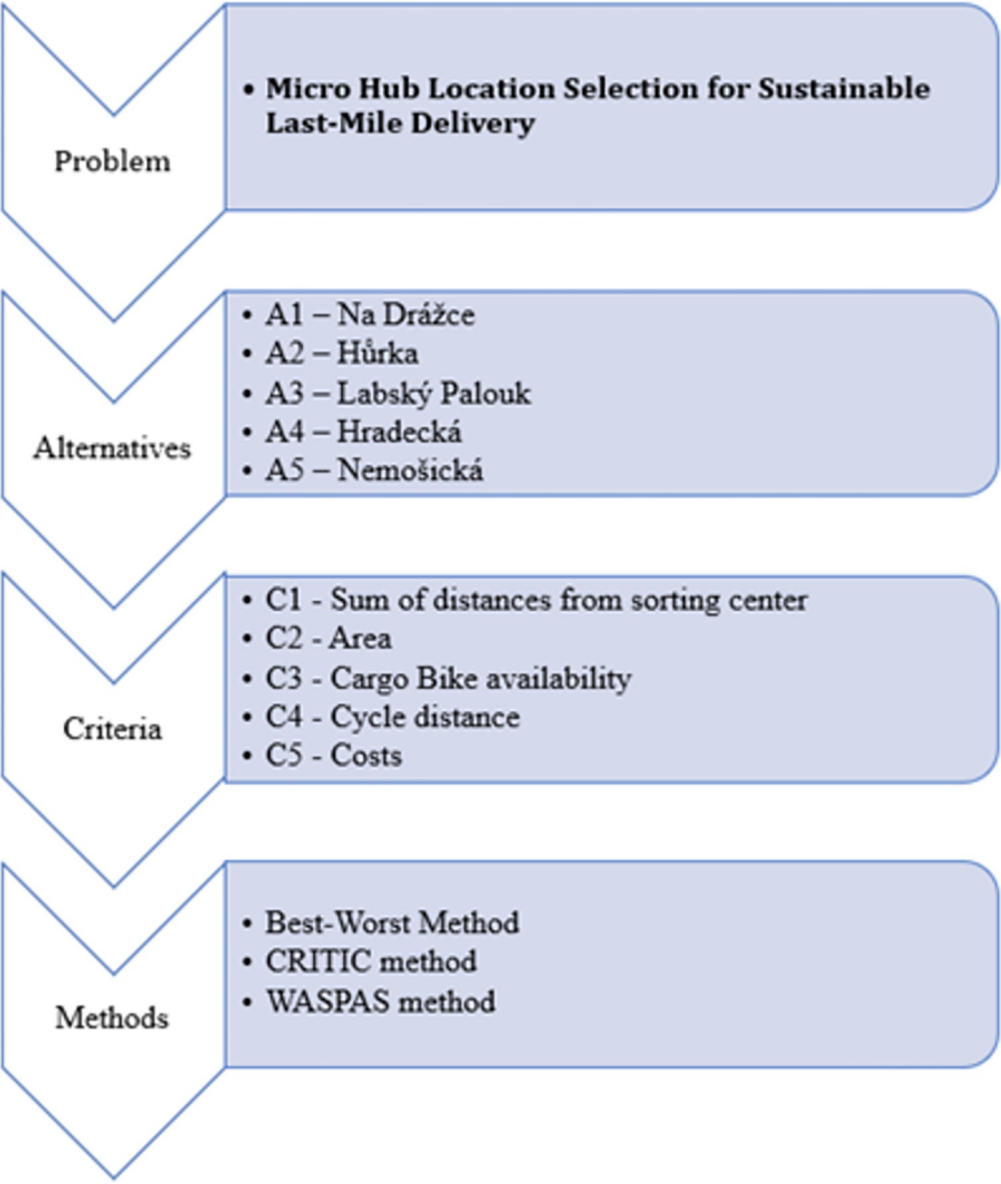
A flowchart of the micro-hub location selection.

This Section shows the application of the previously described methodology on a case study in the context of Pardubice City. The main problem solved in this paper is the micro-hub location selection for sustainable last-mile delivery. There are five alternatives (possible micro-hub locations) compared according to five evaluation criteria. There are three methods coupled to solve the problem mentioned. The first two methods, the Best-Worst Method and the CRITIC method are coupled, and the hybrid criteria weights are obtained. The obtained hybrid weights are integrated within the WASPAS method to rank micro-hub locations in the context of Pardubice city in the Czech Republic. The definition of the problem, criteria and alternatives are elaborated in the further continuation of this section. A flowchart of the problem is presented in Fig 1.

### 4.1 Problem definition

The Pardubice city lies in the Elbe lowlands with a very flat terrain suitable for the use of bicycle traffic. The Capital of the Pardubice district, Pardubice, is an economic, industrial and traffic place with a population of 90,458 inhabitants according to data from the census in 2021. The Pardubice city is a regional city of the Pardubice region. That is why public institutions at the regional level are concentrated in Pardubice. At the same time, Pardubice is an industrial city with several manufacturing companies and a wide range of services. Like any major city, Pardubice faces major problems in the field of transport, not only in peak times. The significance of the problem in the field of transport is also evidenced by the creation of the *Parduplán*—Sustainab–e Urban Mobility Plan. The investigated area of the city center of Pardubice was determined based on information from the Analytical part of *Parduplán* and based on information from carriers. This is an area locally bordered by the rivers *Elbe* and *Chrudimka*, a bypass along the streets of *Kp*. *Jaroš* and *Hlaváčkova*, as well as *17*. *Listopadu* streets, *Masarykovo náměstí* and *Hradecká* streets, which follow each other seamlessly and form the main road. A well-organized last-mile delivery process should be of vital importance for this City.

### 4.2 Definition of alternatives

There are five alternatives that are considered as possible locations for a micro-hub location for the last-mile delivery purpose. Those five possible s were selected from the urbanization plan of Pardubice city. According to the urbanization plan, those possible locations are not utilized by the municipality of Pardubice city. For that reason, the authors saw an opportunity to utilize those locations as the possible alternatives to locate the micro-hub for the last-mile delivery process. The alternatives are further discussed with the academics (experts) from the field of postal traffic and logistics where they agreed that the methodology should be applied to evaluate them. However, the methodology is general and can be applied to any other MCDM problem in city logistics and wider. Nevertheless, the main goal of this paper was to demonstrate the applicability of the methodology and suggest to the Pardubice city possible micro-hub location for the sustainable last-mile delivery process. The possible alternatives (micro-hub locations) described below in Table 6 are compared according to five evaluation criteria.

**Table 6:** Description of the alternatives.

**Alternative 1—Na Drážce**
• Distance from sorting centers–Česká pošta s.p. 3.3 km; Zásilkova 22.6 km; PPL 20.4 km; DPD 3.8 km; DACHSER 30.1 km; Sum of distance 80.2 km.
• Suitability for bicycle transport—Evaluation of routes in terms of cycle routes and traffic density 3.
• The length of the route to the reserved area– 1.7 km.
• Capacity expansion– 4 000 m^2^.
• Construction costs– 800 000 Kč.
**Alternative 2—Hůrka**
• Distance from sorting centers–Česká pošta s.p. 4.2 km; Zásilkova 22 km; PPL 20.7 km; DPD 5.2 km; DACHSER 28 km; Sum of distance 80.1 km.
• Suitability for bicycle transport—Evaluation of routes in terms of cycle routes and traffic density 4.2.
• The length of the route to the reserved area– 3.2 km.
• Capacity expansion– 5 000 m2.
• Construction costs– 550 000 Kč.
**Alternative 3—Labský Palouk**
• Distance from sorting centers–Česká pošta s.p. 1.4 km; Zásilkova 17.7 km; PPL 18.1 km; DPD 7.6 km; DACHSER 26.4 km; Sum of distance 71.2 km.
• Suitability for bicycle transport—Evaluation of routes in terms of cycle routes and traffic density 4.7.
• The length of the route to the reserved area– 2 km.
• Capacity expansion– 2 000 m2.
• Construction costs– 1 250 000 Kč.
**Alternative 4—Hradecká**
• Distance from sorting centers–Česká pošta s.p. 3.2 km; Zásilkova 15 km; PPL 15.4 km; DPD 8 km; DACHSER 23.3 km; Sum of distance 80.1 km.
• Suitability for bicycle transport—Evaluation of routes in terms of cycle routes and traffic density 4.5.
• The length of the route to the reserved area– 1.4 km.
• Capacity expansion– 1 500 m2.
• Construction costs– 1 100 000 Kč.
**Alternative 5—Nemošická**
• Distance from sorting centers–Česká pošta s.p. 3.5 km; Zásilkova 20.2 km; PPL 20.4 km; DPD 5 km; DACHSER 29 km; Sum of distance 80.1 km.
• Suitability for bicycle transport—Evaluation of routes in terms of cycle routes and traffic density 4.2.
• The length of the route to the reserved area– 2.1 km.
• Capacity expansion– 20 000 m2.
• Construction costs– 900 000 Kč.

### 4.3 Definition of evaluation criteria

To select the micro-hub location in Pardubice city, five criteria are considered:

**C**_**1**_**—Sum of di–tance from sorting center**—The first–criterion evaluates the variants in terms of their distance from the logistics centers of individual carriers, which participate in the distribution of shipments to the examined area of Pardubice. The selection of the participating carriers considered the market share of the carriers and their willingness to participate in the *Cyklodepo* project. Some of them are already participating in a similar project in the capital of the Czech Republic—Prague, s–ecifically in the *Cyklodepo* project in Florence, which will soon be expanded by another depot in *Anděl*. The sum of distances to the micro-hub was thus examined for the carriers *Česká pošta s*.*p*., *Zásilkovna*.*cz*, *PPL CZ s*.*r*.*o*., *DPD group* and *DACHSER Czech Republic a*.*s*.

**C**_**2**_ **–Area*—***The secon–criterion evaluates the variants in terms of their area with regard to the possibilities of spreading the micro-hub: 1) other services–parcel locker, electric car, etc.; 2) other interested parties—with rega–d to the possibility of introducing entry restrictions for the examined area; 3) expansion of the micro-hub location area—with rega–d to the further development of transport, suppression of externalities, costs. Following the above-mentioned, it is necessary to think about the expansion of the depot and look for areas that will allow further expansion without greater costs or the need to change the micro-hub location.

**C**_**3**_ **–Cargo bike availability***—*The cargo–bike transport is one of the promoted alternatives to road transport. However, its main disadvantage is security. The consequences of a cyclist’s collisio’ with a road vehicle are often fatal. Such consequences can only be avoided by separating the infrastructure for road vehicles and bicycles, which leads to high costs. In this way, cities approach alternative solutions, where separate bicycle paths are built on exposed parts, and in other parts bicycle paths are run along local roads with a low level of traffic. The individual routes from the micro-hub to the investigated area were thus evaluated in terms of their guidance along cycle paths, low-traffic roads, or medium-high-traffic roads. The route was divided by meters and the ratio of the route led along the given cycle route/road was multiplied by a coefficient from 1 to 5, where 5 was the rating of the separately led cycle route and the rating 1 for the road with high traffic.

**C**_**4**_ **–Cycle distance–**With regard to minimizing the length of the route and the route recommended for cyclists from the maps provided by the City by Bike Magazine, optimal routes from the micro-hub on the border of the researched area in the centre of Pardubice were determined.

**C**_**5**_ –**Costs**–Another important criterion examined was the costs. This criterion was determined by experts in the field. This is the sum of the costs of providing the necessary needs for the formation and operation of the micro-hub. These include strengthening the area used, building social facilities for employees of micro-hub (toilets, showers), fencing the land for the safety of consignments and employees, and finally, building an electrical connection with regard to charging stations for cargo bikes, electric vehicles, etc. When selecting localities, only those plots of land that are owned by the city of Pardubice were selected. In the *Cyklodepa* project in Florence (Prague), localities were selected with regard to the roofing. Due to the fact that no suitable area was found in the city of Pardubice, it would be necessary to solve the roofing with the same parameters in selected areas, therefore these costs were not included.

## 5. Results and discussion

This section presents the results obtained by the methodology proposed.

### 5.1 Experimental results

Five estimated micro-hub locations in the context of Pardubice city are “Na Dražce” (A_1_), “Hůrka” (A_2_), “Labský Palouk” (A_3_), “Hradecká” (A_4_), and “Nemošická” (A_5_). Five criteria (sum of distance from sorting center (C_1_), area (C_2_), cargo bike availability (C_3_), cycle distance (C_4_), and costs (C_5_)) influencing the micro-hub location selection process were considered. Three Postal Traffic Engineers participated as the experts in the case study (Table 7).

**Table 7:** The information about the experts.

Expert	Gender	Qualifications	Experience
**Expert 1**	Male	Ph.D.	Employee in the Postal Branch with 8 years of experience in distribution
**Expert 2**	Male	Ph.D.	Associate professor at the Postal Traffic Department with 12 years of experience
**Expert 3**	Male	Ph.D.	Associate professor at the Postal and Logistics Department with 10 years of experience

The invited experts were interviewed by telephone due to the COVID-19 outbreak. They were sorted out based on their qualifications and experience. In addition, the criteria and alternatives that influence the micro-hub location selection problem have been defined in collaboration with experts.

The first two methods applied to evaluate the criteria weights for the micro-hub location problem were the Best-Worst and the CRITIC methods. Those two methods are coupled into the hybrid one and utilized within the WASPAS method to obtain the final rank of the possible micro-hub locations. The results obtained by the best-worst method are presented in the following tables (Tables 8–11).

**Table 8:** Initial criteria for a decision-making process.

Criteria	Criterion 1	Criterion 2	Criterion 3	Criterion 4	Criterion 5
**Names of Criteria**	Cycle Distance [m]	Area [m^2^]	Cargo Bike Availability	Costs [CZK]	Sum distance [km]

**Table 9:** Comparison of the best alternative to others.

Best to Others	Cycle Distance [m]	Area [m^2^]	Cargo Bike Availability	Costs [CZK]	Sum distance [km]
**Area [m^2^]**	2	1	3	5	5

**Table 10:** Comparison of other alternatives to the worst one.

Others to the Worst	Cycle Distance [m]	Area [m^2^]	Cargo Bike Availability	Costs [CZK]	Sum distance [km]
**Sum distance [km]**	5	7	4	2	1

**Table 11:** Final criteria weights calculated by the best-worst method.

	Cycle Distance [m]	Area [m^2^]	Cargo Bike Availability	Costs [CZK]	Sum distance [km]
**Criteria Weights (Wbw)**	0.2523	0.4140	0.1682	0.1009	0.0647
***Ksi = 0*.*0906***					

When it comes to CRITIC method, the following results were obtained (Tables 12–15).

**Table 12:** The initial CRITIC decision-making matrix.

	Cycle Distance [m]	Area [m^2^]	Cargo Bike Availability	Costs [CZK]	Sum distance [km]
**A_1_**	1 700	4 000	3	800 000	80.2
**A_2_**	3 200	5 000	4.2	550 000	80.1
**A_3_**	2 000	2 000	4.7	1 250 000	71.2
**A_4_**	1 400	1 500	4.5	1 100 000	64.9
**A_5_**	2 100	4 000	4.2	900 000	77.9
**Criteria type**	min	max	max	min	min
**Best**	1 400	5 000	4.7	550 000	64.9
**Worst**	3 200	1 500	3	1 250 000	80.2

**Table 13:** Normalization of the initial decision-making matrix with the standard deviation ỽj.

	Cycle Distance [m]	Area [m^2^]	Cargo Bike availability	Costs [CZK]	Sum distance [km]
**A_1_**	0.8333	0.7143	0.0000	0.6429	0.0000
**A_2_**	0.0000	1.0000	0.7059	1.0000	0.0065
**A_3_**	0.6667	0.1429	1.0000	0.0000	0.5882
**A_4_**	1.0000	0.0000	0.8824	0.2143	1.0000
**A_5_**	0.6111	0.7143	0.7059	0.5000	0.1503
**ỽj**	0.3797	0.4238	0.3889	0.3866	0.4358

**Table 14:** Formulation of the mxm criteria matrix.

	Cycle Distance [m]	Area [m^2^]	Cargo Bike availability	Costs [CZK]	Sum distance [km]
**Cycle Distance [m]**	1.0000	-0.7350	-0.1173	-0.7002	0.6017
**Area [m^2^]**	-0.7350	1.0000	-0.5176	0.9217	-0.9470
**Cargo Bike availability**	-0.1173	-0.5176	1.0000	-0.5072	0.6368
**Costs [CZK]**	-0.7002	0.9217	-0.5072	1.0000	-0.7815
**Sum distance [km]**	0.6017	-0.9470	0.6368	-0.7815	1.0000

**Table 15:** Final criteria weights calculated by the CRITIC method.

	Sum by rows	ỽj	Hj	Criteria Weights (Wc)
**Cycle Distance [m]**	4.9508	0.3797	1.8796	0.1921
**Area [m^2^]**	5.2779	0.4238	2.2367	0.2286
**Cargo Bike availability**	4.5054	0.3889	1.7520	0.1791
**Costs [CZK]**	5.0672	0.3866	1.9592	0.2002
**Sum distance [km]**	4.4901	0.4358	1.9566	0.2000

Hybrid criteria weights are further obtained by combining the criteria weights obtained by the Best-Worst Method and the CRITIC one. The coupling those two methods is calculated by the following Equation:

HybridWeight(Wh)=0.5·Wbw+0.5·Wc (14)

where (*Wh*) represents the hybrid weight

The obtained hybrid criteria weights are presented in Table 16.

**Table 16:** Hybrid criteria weights.

	CRITIC Weights—Wc	BWM Weights—Wbw	Hybrid Weights–Wh
**Cycle Distance [m]**	0.1921	0.2523	0.2222
**Area [m^2^]**	0.2286	0.4140	0.3213
**Bicycle Availability**	0.1791	0.1682	0.1736
**Costs [CZK]**	0.2002	0.1009	0.1506
**Sum distance [km]**	0.2000	0.0647	0.1323

The calculated hybrid criteria weights were utilized within the WASPAS method where the final rank of the micro-hub locations was obtained. The results of the WASPAS method are presented below (Tables 17–20).

**Table 17:** The initial input data processed by the WASPAS method.

Alternative/Criteria	Cycle Distance [m]	Area [m^2^]	Cargo Bike availability	Costs [CZK]	Sum distance [km]
**A_1_**	1 700	4 000	3	800000	80.2
**A_2_**	3 200	5 000	4.2	550 000	80.1
**A_3_**	2 000	2 000	4.7	1250000	71.2
**A_4_**	1 400	1 500	4.5	1100000	64.9
**A_5_**	2 100	4 000	4.2	900000	77.9
	0.2222	0.3213	0.1736	0.1506	0.1323
**Criteria type**	min	max	max	min	min
	1400	5000	4.7	550000	64.9

**Table 18:** Normalization of the input data.

	Cycle Distance [m]	Area [m^2^]	Cargo Bike availability	Costs [CZK]	Sum distance [km]	Weighted Product Method (WPM)
**A_1_**	0.8235	0.8000	0.6383	0.6875	0.8092	4.7311
**A_2_**	0.4375	1.0000	0.8936	1.0000	0.8102	4.7854
**A_3_**	0.7000	0.4000	1.0000	0.4400	0.9115	4.5403
**A_4_**	1.0000	0.3000	0.9574	0.5000	1.0000	4.5726
**A_5_**	0.6666	0.8000	0.8936	0.6111	0.8331	4.7300

**Table 19:** Weighted decision-making matrix.

	Cycle Distance [m]	Area [m^2^]	Cargo Bike availability	Costs [CZK]	Sum distance [km]	Simple Additive Weighting Method (SAW)
**A_1_**	0.1829	0.2570	0.1108	0.1035	0.1071	0.7614
**A_2_**	0.0972	0.3213	0.1551	0.1506	0.1072	0.8314
**A_3_**	0.1555	0.1285	0.1736	0.0663	0.1206	0.6445
**A_4_**	0.2222	0.0964	0.1662	0.0753	0.1323	0.6924
**A_5_**	0.1481	0.2570	0.1551	0.0920	0.1102	0.7626

**Table 20:** The final rank of the alternatives according to the parameter λ.

	λ = 0	λ = 0.1	λ = 0.2	λ = 0.3	λ = 0.4	λ = 0.5	λ = 0.6	λ = 0.7	λ = 0.8	λ = 0.9	λ = 1
**A_1_**	4.7311	4.3342	3.9372	3.5402	3.1433	2.7463	2.3493	1.9523	1.5554	1.1584	0.7614
**A_2_**	4.7854	4.3900	3.9946	3.5992	3.2038	2.8084	2.4130	2.0176	1.6222	1.2268	0.8314
**A_3_**	4.5403	4.1507	3.7611	3.3716	2.9820	2.5924	2.2028	1.8133	1.4237	1.0341	0.6445
**A_4_**	4.5726	4.1845	3.7965	3.4085	3.0205	2.6325	2.2445	1.8564	1.4684	1.0804	0.6924
**A_5_**	4.7300	4.3332	3.9365	3.5397	3.1430	2.7463	2.3495	1.9528	1.5560	1.1593	0.7626

The final rank of the alternatives is presented in Table 20. To test the obtained results, the sensitivity analysis was performed, where the parameter λ was varied between intervals 0 to 1.

**Fig 2:**
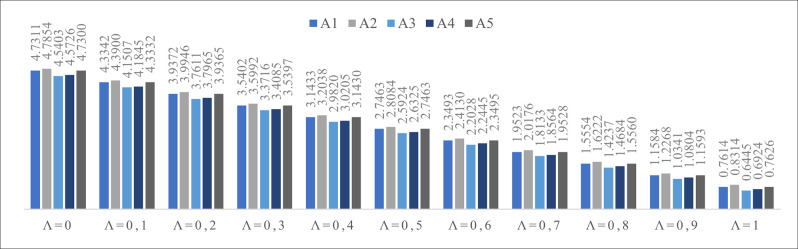
Rank of the alternatives when λ is between intervals 0 and 1 (Source: Authors).

When λ is equal to 1, only the Simple Additive Weighting (SAW) method affects ranking the alternatives. On the contrary, when λ is equal to 0, only the Weighted Product Method (WPM) affects ranking the alternatives. In all other cases, both the SAW and the WPM methods affect the ranking of alternatives. Fig 2 shows the final rank of the considered alternatives according to different values of the parameter λ.

### 5.2 Comparative analysis

**Fig 3:**
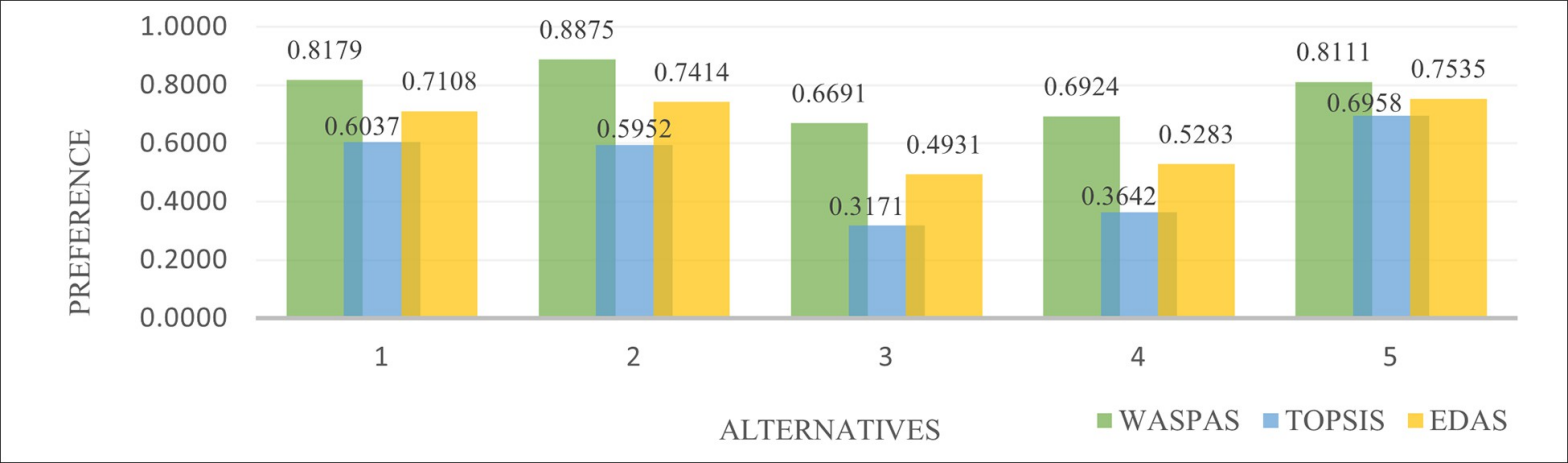
The comparative analysis of the hybrid BWM-CRITIC-WASPAS method with TOPSIS and EDAS (Source: Authors).

A comparative analysis, as one of the most important parts of the research paper, is used as an indicator of the reliability of the methodology. In this paper, the comparative analysis was performed to check the reliability of the results obtained through the hybrid BWM-CRITIC-WASPAS method for micro-hub location selection. The micro-hub location selection was solved with two previously proposed methods, TOPSIS [95] and EDAS [64]. The result of the comparative analysis is schematically presented in Fig 3.

The Fig 3 shows that the proposed hybrid BWM-CRITIC-WASPAS method ranks the alternatives in the following ranking order: *“Hůrka” (A*_*2*_*)> “Na Dražce” (A*_*1*_*)> “Nemošická” (A*_*5*_*)> “Hradecká” (A*_*4*_*)> “Labský Palouk” (A*_*3*_*)*. Along with the proposed method, the EDAS method ranks the alternatives in the following order: *A*_*5*_*> A*_*2*_*> A*_*1*_ *>A*_*4*_*> A*_*3*_. According to the TOPSIS method, there is the following rank of the alternatives: *“Nemošická” (A*_*5*_*)> “Na Dražce” (A*_*1*_*)> “Hůrka” (A*_*2*_*)> “Hradecká” (A*_*4*_*)> “Labský Palouk” (A*_*3*_*)*. It should be noticed that the TOPSIS method ranks the *A*_*4*_ and *A*_*3*_ as the previous two methods, while Alternative 2 (*“Hůrka”*) was ranked at the third place which is still a high preference for this alternative. The best alternative as a possible micro-hub location according to TOPSIS was Alternative 5 (*“Nemošická”)* while Alternative 1 (*“Na Dražce”*) was ranked the second place. Therefore, the Alternative 2 (*“Hůrka”*) was identified as the best possible solution for micro-hub location for sustainable last-mile delivery. This alternative has 4.2 km from *Česká pošta*, 22 km from *Zásilkova*, 20.7 km *PPL*, 5.2 km from *DPD*, 28 km from *DACHSER*, and the overall sum of distance 80.1 km. Regarding the capacity expansion, it is equal to 5000 m^2^, with the total construction costs worth 550.000 Kč. The length of the route to the reserved area is 3.2 km. The Alternative 1 (*“Na Dražce”*) was identified as the second best for micro-hub location for sustainable last-mile delivery. When it comes to Alternative 1, this alternative has 3.3 km from *Česká pošta*, 22.6 km from *Zásilkova*, 20.4 km *PPL*, 3.8 km from *DPD*, 30.1 km from *DACHSER*, and the overall sum of distance 80.2 km. Regarding the capacity expansion, it is equal to 4000 m^2^, with the total construction costs worth 800.000 Kč. The length of the route to the reserved area is 1.7 km.

Regarding the facility location problem, Rosenberg et al. [13] introduced the shared Micro-Depot (MD) network for last-mile logistics. They evaluated the success of shared micro-depot networks, considering economic, environmental, and social aspects. They concluded that when the MD’s facility was operated by a third party, it allows logistics service providers to operate more efficiently. Karaşan el al. [92] selected the best location for a charging station in Istanbul, Turkey. They considered the possibility where to locate the Electric Vehicle charging station, the *Anatalia side* or the *European side* of Istanbul. Nine alternatives were compared according to four evaluation criteria with the sub criteria. As the best location for the charging station, they identified *Kadiköy Dock District*.

As may be noticed, the location selection problem has been becoming more and more popular and should have an increasing trend in the future sustainable urban life.

### 5.3 Managerial implications

Since the LMD process is one of the crucial city logistics issues, managers and postal traffic engineers must take a lot of effort to make it as efficient and sustainable as possible. Choosing the right micro hub location for the needs of the LMD is a problem of vital importance and can significantly affect the quality of delivery, operating costs, better organization of delivery in the city, as well as a higher degree of customer satisfaction. When deciding the micro-hub localization, many conflicting criteria should be considered and evaluated by some of the MCDM methods including the experts’ assistance. The wrong choice of the micro-hub location can have a negative impact on the postal companies and their final customers in many aspects such as high costs, low quality of delivery, low customer satisfaction, the crowd in the cities, etc. Managers must carefully monitor and assess the current and future trends in the LMD process and ensure the highest possible quality to sustain the existing and gain new customers in cities.

### 5.4 Scientific implications

The micro-hub location problem is a relatively new kind of problem for the purpose of last-mile delivery. From the scientific point of view, this kind of problem pretends to be further discussed among scientists, since it is of huge importance for sustainable last-mile delivery, especially in urban areas. More and more modern and innovative last-mile delivery solutions are emerging (drone delivery, cargo bike delivery, electric vehicle delivery, autonomous vehicles), and along with that, it is necessary to put lots of effort into both the practical and scientific spheres. The intention of this study is to encourage other scientists in this field to give as much contribution as possible to make the last-mile delivery process become more effective, sustainable, and make cities better places for future life.

## 6. Conclusions

The Hybrid BWM-CRITIC-WASPAS multi-criteria decision-making approach is demonstrated in this paper. Its main contribution is methodological and practical ones. Considering methodological contribution, for the first time the hybrid BWM-CRITIC-WASPAS MCDM approach is proposed to solve the micro-hub location selection problem. The Best-Worst and CRITIC methods were effectively coupled to obtain hybrid criteria weights that were further integrated within the WASPAS method to rank the selected micro-hub locations. The formulated hybrid BWM-CRITIC-WASPAS approach was applied in real-life, in the context of Pardubice city, which should be considered as a practical contribution.

The advantages of the introduced real-life case study are: *i)* The usefulness of the introduced approach has been demonstrated. The proposed BWM-CRITIC-WASPAS method is general and can be applied not only to rank the micro-hub locations for LMD but also to any other MCDM problem. The method combines the objective and subjective methods the find the criteria importance, which is a way better than applying just one (for instance, subjective method). We all strive to reduce the subjectivity to the lowest possible level to have more precise results in a decision-making process. The applied BWM-CRITIC-WASPAS method is particularly suitable in situations when the input data are given in the form of crisp values. *ii)* The high level of robustness of the introduced hybrid BWM-CRITIC-WASPAS has been verified by sensitivity analysis since the rank of the micro-hub locations is unchangeable to variations in parameter λ. *iii)* The high level of reliability of the hybrid BWM-CRITIC-WASPAS is confirmed by the comparative analysis with the TOPSIS and EDAS methods.

The introduced hybrid BWM-CRITIC-WASPAS approach for the micro-hub location selection generates the following ranking order: “Hůrka”- A2 > “Na Dražce”—A1 > “Nemošická”—A5 > “Hradecká”—A4 > “LabskýPalouk”-A3. The proposed hybrid BWM-CRITIC-WASPAS approach suggested the Alternative 2 (“Hůrka”) as the most suitable one for the last-mile delivery process. The alternative has 4.2 km from Česká pošta, 22 km from Zásilkova, 20.7 km PPL, 5.2 km from DPD, 28 km from DACHSER, and the overall sum of distance 80.1 km. Regarding the capacity expansion, it is equal to 5000 m2, with the total construction costs worth 550.000 Kč. The length of the route to the reserved area is 3.2 km. On the other side, the Alternative 3 (“Labský Palouk”) was the worst-ranked one.

There are some limitations of this article that should be used as a possible extension: 1) The proposed hybrid BWM-CRITIC-WASPAS method only deals with the crisp values, and it is not integrated with the fuzzy logic since it deals with uncertainty. 2) Due to the COVID-19 pandemic, only three experts were participated in creating a real-life case study.

Some of the future research directions should be: 1) to upgrade the methodology into the fuzzy environment with various types of fuzzy numbers; 2) to include many more experienced experts in a decision-making process; 3) To apply the methodology in bigger cities considering more interrelated criteria; 4) After deciding on the micro-hub location, a possible future direction should be to evaluate and select cargo bikes that should be appropriate for the LMD process. In the future, the last-mile delivery process in the cities will be of vital importance to make a more modern and sustainable life.

This research was part of the project „CK01000032 –Sustainable Urban Mobility Plans, E-commerce, and Smart City Logistics“.

## Supporting information

S1 Dataset (DOCX)
