# Supplementary material for: Micro-hub location selection for sustainable last-mile delivery
Source: PLoS One. 2022 Jul 5;17(7):e0270926. doi: 10.1371/journal.pone.0270926 (PMC9255753; doi:10.1371/journal.pone.0270926)
Supplement: S1 Dataset — (DOCX) [file pone.0270926.s001.docx]

**MINIMAL DATA SET DEFINITION - Micro-Hub Location Selection for Sustainable Last-Mile Delivery**

For the purpose of this study, we enclose the input data that we processed throughout the methodologies applied. The input data are structured and disclosed in Table 1.

Table 1. The input data used in the micro-hub location selection problem

| Alternative 1 - Na Drážce |
| --- |
| - Distance from sorting centers – Česká pošta s.p. 3.3 km; Zásilkova 22.6 km; PPL 20.4 km; DPD 3.8 km; DACHSER 30.1 km; Sum of distance 80.2 km. |
| - Suitability for bicycle transport - Evaluation of routes in terms of cycle routes and traffic density 3. |
| - The length of the route to the reserved area – 1.7 km. |
| - Capacity expansion – 4 000 m^2^. |
| - Construction costs – 800 000 Kč. |
| Alternative 2 - Hůrka |
| - Distance from sorting centers – Česká pošta s.p. 4.2 km; Zásilkova 22 km; PPL 20.7 km; DPD 5.2 km; DACHSER 28 km; Sum of distance 80.1 km. |
| - Suitability for bicycle transport - Evaluation of routes in terms of cycle routes and traffic density 4.2. |
| - The length of the route to the reserved area – 3.2 km. |
| - Capacity expansion – 5 000 m^2^. |
| - Construction costs – 550 000 Kč. |
| Alternative 3 - Labský Palouk |
| - Distance from sorting centers – Česká pošta s.p. 1.4 km; Zásilkova 17.7 km; PPL 18.1 km; DPD 7.6 km; DACHSER 26.4 km; Sum of distance 71.2 km. |
| - Suitability for bicycle transport - Evaluation of routes in terms of cycle routes and traffic density 4.7. |
| - The length of the route to the reserved area – 2 km. |
| - Capacity expansion – 2 000 m^2^. |
| - Construction costs – 1 250 000 Kč. |
| Alternative 4 - Hradecká |
| - Distance from sorting centers – Česká pošta s.p. 3.2 km; Zásilkova 15 km; PPL 15.4 km; DPD 8 km; DACHSER 23.3 km; Sum of distance 80.1 km. |
| - Suitability for bicycle transport - Evaluation of routes in terms of cycle routes and traffic density 4.5. |
| - The length of the route to the reserved area – 1.4 km. |
| - Capacity expansion – 1 500 m^2^. |
| - Construction costs – 1 100 000 Kč. |
| Alternative 5 - Nemošická |
| - Distance from sorting centers – Česká pošta s.p. 3.5 km; Zásilkova 20.2 km; PPL 20.4 km; DPD 5 km; DACHSER 29 km; Sum of distance 80.1 km. |
| - Suitability for bicycle transport - Evaluation of routes in terms of cycle routes and traffic density 4.2. |
| - The length of the route to the reserved area – 2.1 km. |
| - Capacity expansion – 20 000 m^2^. |
| - Construction costs – 900 000 Kč. |

The input data set utilized in designing Figure 4 in the Manuscript is presented in Table 2.

Table 2. The Input Data Set used to perform the Sensitivity Analysis

|  | λ=0 | λ=0.1 | λ=0.2 | λ=0.3 | λ=0.4 | λ=0.5 | λ=0.6 | λ=0.7 | λ=0.8 | λ=0.9 | λ=1 |
| --- | --- | --- | --- | --- | --- | --- | --- | --- | --- | --- | --- |
| A_1_ | 4.7311 | 4.3342 | 3.9372 | 3.5402 | 3.1433 | 2.7463 | 2.3493 | 1.9523 | 1.5554 | 1.1584 | 0.7614 |
| A_2_ | 4.7854 | 4.3900 | 3.9946 | 3.5992 | 3.2038 | 2.8084 | 2.4130 | 2.0176 | 1.6222 | 1.2268 | 0.8314 |
| A_3_ | 4.5403 | 4.1507 | 3.7611 | 3.3716 | 2.9820 | 2.5924 | 2.2028 | 1.8133 | 1.4237 | 1.0341 | 0.6445 |
| A_4_ | 4.5726 | 4.1845 | 3.7965 | 3.4085 | 3.0205 | 2.6325 | 2.2445 | 1.8564 | 1.4684 | 1.0804 | 0.6924 |
| A_5_ | 4.7300 | 4.3332 | 3.9365 | 3.5397 | 3.1430 | 2.7463 | 2.3495 | 1.9528 | 1.5560 | 1.1593 | 0.7626 |

The input data set utilized in designing Figure 5 in the Manuscript is presented in Table 3.

Table 3. The Input Data Set used to perform the Comparative Analysis

|  | WASPAS | TOPSIS | EDAS |
| --- | --- | --- | --- |
| A_1_ | 0.8179 | 0.6037 | 0.7108 |
| A_2_ | 0.8875 | 0.5952 | 0.7414 |
| A_3_ | 0.6691 | 0.3171 | 0.4931 |
| A_4_ | 0.6924 | 0.3642 | 0.5283 |
| A_5_ | 0.8111 | 0.6958 | 0.7535 |
